# Research on the Properties and Low Cycle Fatigue of Sc-Modified AA2519-T62 FSW Joint

**DOI:** 10.3390/ma13225226

**Published:** 2020-11-19

**Authors:** Robert Kosturek, Lucjan Śnieżek, Janusz Torzewski, Tomasz Ślęzak, Marcin Wachowski, Ireneusz Szachogłuchowicz

**Affiliations:** Faculty of Mechanical Engineering, Military University of Technology, 2 gen. S. Kaliskiego St., 00-908 Warsaw, Poland; lucjan.sniezek@wat.edu.pl (L.Ś.); janusz.torzewski@wat.edu.pl (J.T.); tomasz.slezak@wat.edu.pl (T.Ś.); marcin.wachowski@wat.edu.pl (M.W.); ireneusz.szachogluchowicz@wat.edu.pl (I.S.)

**Keywords:** aluminum, friction stir welding, microstructure, mechanical properties, residual stresses, fatigue, fracture

## Abstract

The aim of this research was to examine the mechanical and fatigue properties of friction stir welded Sc-modified 5 mm thick AA2519-T62 extrusion. The joint was obtained using the following parameters: 800 rpm tool rotation speed, 100 mm/min tool traverse speed, 17 kN axial, and MX Triflute as a tool. The investigation has involved microstructure observations, microhardness distribution analysis, tensile test with digital image correlation technique, observations of the fracture surface, measurements of residual stresses, low cycle fatigue testing, and fractography. It was stated that the obtained weld is defect-free and has joint efficiency of 83%. The failure in the tensile test occurred at the boundary of the thermo-mechanically affected zone and stir zone on the advancing side of the weld. The residual stress measurements have revealed that the highest values of longitudinal stress are localized at the distance of 10 mm from the joint line with their values of 124 MPa (the retreating side) and 159 MPa (the advancing side). The results of low cycle fatigue testing have allowed establishing of the values of the cyclic strength coefficient (k′ = 504.37 MPa) and cyclic strain hardening exponent (n′ = 0.0068) as well as the factors of the Manson–Coffin–Basquin equation: the fatigue strength coefficient σ′_f_ = 462.4 MPa, the fatigue strength exponent b = −0.066, the fatigue ductility coefficient ε′_f_ = 0.4212, and the fatigue ductility exponent c = −0.911.

## 1. Introduction

Friction stir welding (FSW) is a very efficient technology in the production of aluminum alloy joints, as it provides a number of advantages over traditional fusion welding such as the lower temperature of the joining process [1,2,3]. This particular factor is crucial when it comes to welding of high-strength aluminum alloys, which are mainly precipitated-hardening materials (2XXX and 7XXX series) and their specific strength is an effect of thermally unstable precipitates (e.g., Guinier–Preston zones, θ′ phase) [4,5,6,7]. Considering the production of efficient welds of these materials, the losses in hardening must be taken into account due to the thermal affection of the joining process on the workpiece. In this paper, AA2519-T62 armor grade aluminum is taken under investigation in terms of its friction stir-welded joint properties. This alloy contains 5.3–6.4% copper and is subjected to the precipitation hardening process, and thus, acquires high specific strength, which makes it a very desirable material in terms of aerospace, automotive, and military applications [8,9,10]. It is noteworthy that the investigated alloy is a modification of AA2519 characterized by the addition of scandium and zirconium, which improves its mechanical properties and resistance to elevated temperature in the form of higher recrystallization temperature [9,11,12,13]. Although friction stir welding is a suitable technology for joining AA2519, it still significantly influences its microstructure and (at the same time) mechanical properties. The changes in the alloy properties include grain refinement in the stir zone, dissolution and coarsening of strengthening phase, and generation of residual stresses [2,14,15]. In recent years, friction stir welded joints of high-strength aluminum alloys (including AA2519) were the subject of several studies, focused mostly on some microstructural aspects of welds, their basic mechanical properties, heat distribution, and optimization of welding parameters (also using artificial intelligence) [16,17,18,19,20,21]. Despite major progress in the mentioned fields of study, the issue of the most complex mechanical properties of FSW joints received disproportionately less attention, and the aspects providing their structural integrity (corrosion, fatigue, dynamic load) are still a gap in the current state of knowledge, especially for less popular alloys such as AA2519. All changes caused by the FSW process generate discontinuity in the welded workpieces: differences in the grain size, in the distribution of the strengthening phase precipitates (for heat-treated aluminum alloys) and the residual stresses, and the evolution of the surface geometry and roughness, that result in local stress concentration during load-carrying operating of a welded element [22,23,24,25,26,27,28,29]. In these terms, several studies have been undertaken to investigate the fatigue properties of 2XXX aluminum alloy FSW joints in the past few years. Xu et al. investigated the low cycle fatigue (LCF) properties of 2219-T62 alloy under various FSW parameters and cooling conditions (air/water) and reported that fatigue life decreased with increasing welding speed (60 to 200 mm/min) and that it was independent of the tool rotation speed (300 to 1000 rpm) [30]. The same study showed that the joints obtained by water-cooled FSW have higher stress amplitudes and fatigue lives. Sun et al. performed research on fatigue modeling and life prediction of AA2219-T6 FSW joints; focusing on the microstructural aspects of the joints supported by the results of micro tensile tests the authors proposed a model with good accuracy [31]. Vuherer et al. analyzed fatigue behavior of AA2024 T-351 aluminum alloy sheets joined by FSW with various parameters and based on the results they described the relationship between welding velocity and basic fatigue properties focusing on the issue of heat input and coarse of microstructure [32]. Based on our own research, we can state that post-weld heat treatment decreases the fatigue strength of AA2519 FSW joints significantly [33] and the optimal welding parameters for welding of AA2519-T62 lie within the range of 600–800 rpm tool rotation speed and 100 mm/min welding velocity, giving relatively high joint efficiency (around 80%) and good fatigue properties [34]. Considering the issue of high complexity, which is evident in the behavior of FSW joints during cyclic loading in the low cycle fatigue regime, a number of factors have to be taken under examination, susceptible to the high plastic strain amplitude (e.g., heat-affected zone, low-hardness zone, stir zone/thermo-mechanically affected zone interface) [34,35,36,37]. LCF is important from the point of view of estimating the durability of structural elements at the design stage of the structure, during its operation, as well as in the analysis of the assessment of the structure life (and the possibility of its extension). The aim of this paper is to investigate the LCF properties of 5 mm-thick AA2519-T62 FSW joints together with basic mechanical properties, microstructure analysis, and residual stresses measurements.

## 2. Material and Experimental Procedures

The investigated material was AA2519-T62 extrusion with dimensions of 5 × 80 × 250 mm. The precipitation hardening process was performed by solution treatment (530 °C/2 h + cooling in cold water) and subsequent artificial aging (165 °C/10 h). The chemical composition and mechanical properties of the alloy are presented in Table 1 and Table 2, respectively.

**Figure 1:**
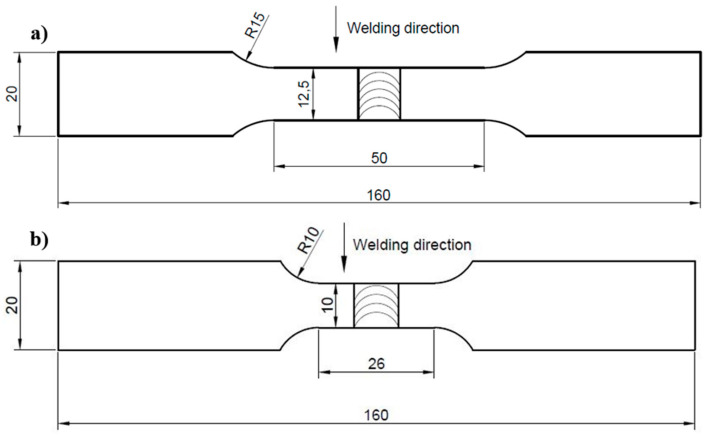
The scheme of sample for (**a**) tensile test and (**b**) low cycle fatigue testing.

Prior to the joining process, the workpieces to be welded were ground and cleaned with isopropanol. The FSW was conducted with an ESAB FSW Legio 4UT (ESAB, Gothenburg, Sweden) machine with the applied axial force of 17 kN. The tool rotation speed and tool traverse speed were equal to 800 rpm and 100 mm/min, respectively. The type of tool used was an MX Triflute (ESAB, Gothenburg, Sweden) and the tilt angle was set to 2°. These parameters were selected based on the previous study performed by authors [34]. The obtained joint was sectioned perpendicular to the welding direction. The metallographic preparation involved cutting a sample from the joint, mounting it in resin, grinding it with abrasive papers of 80, 320, 500, 800, 1200, 2400, and 4000 gradations, and polishing with diamond pastes (gradations of 3 and 1 μm). The prepared sample was etched with Keller reagent (20 mL H_2_O + 5 mL 63% HNO_3_ + 1 mL 38% HCl + one drop of 40% HF) for about 10 s. The microstructure observations have been conducted on the Olympus LEXT OLS 4100 digital light microscope (Olympus, Tokyo, Japan). The Vickers microhardness (Struers Copenhagen, Denmark) of the weld was measured on its polished cross-section by applying 0.98 N load in accordance with the EN ISO 6507 standard. The distribution of microhardness was obtained for the top, middle, and bottom parts of the cross-section of the weld: 0.8, 2.5, and 4.4 mm from the face of the joint, respectively. The basic mechanical properties of the joint were examined by tensile testing according to ASTM standard E8/E8M–13a [38]. Tensile tests were carried out on an Instron 8802 MTL universal testing machine (Instron, Norwood, MA, USA) with WaveMatrix computer software (Instron, Norwood, MA, USA). Additionally, the tensile test was supported by digital image correlation (DIC) in order to examine the local strain in the welded joint zone. For DIC, the Dantec Q-400 optical system (Dantec Dynamics GmbH, Ulm, Germany) was used, and the obtained data were processed with Istra4D 4.4.7 software (version 4.4.7). The fatigue testing was conducted on an Instron 8802 servohydraulic fatigue testing system according to ASTM E606/E606M standard. To measure the value of strain during testing a 2520-603 dynamic extensometer (Instron, Norwood, MA, USA) with a gauge length of 25 mm was used. The fatigue tests were carried out on five various levels of total strain amplitude: 0.35%, 0.4%, 0.5%, 0.6%, and 0.8% with strain ratio R = 0.1. For each level, three samples were examined. The fracture surfaces of tensile and fatigue samples were analyzed on the scanning electron microscope (SEM) Jeol JSM-6610 (Jeol, Tokyo, Japan). The samples for tensile and fatigue testing were prepared with the geometry presented in Figure 1.

**Figure 2:**
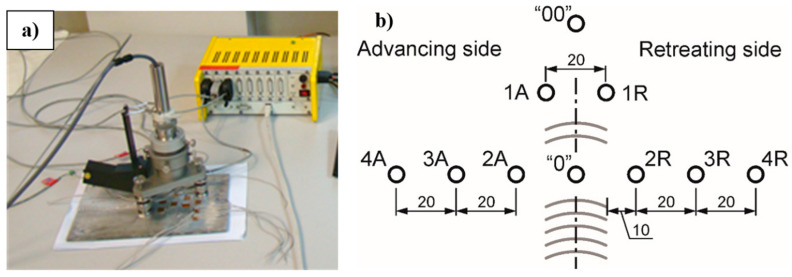
The view of a measuring stand with RS-200 system with the tensometer bridge ESAM Traveler Plus (**a**) and the location of numbered measuring points on the plate with friction stir welding (FSW) joint (**b**).

The values of residual stresses were established by the hole-drilling method which is described in ASTM E 837 in detail [39]. Because of the thickness of the plate (5 mm), the blind-hole procedure was used. In this approach, the hole is drilled at the depth of 2 mm and the released strains are measured by the specially designed rosette. The accurate location of the hole in the center point of the rosette was ensured by using the VPG Micro-Measurement System RS-200 milling guide (Micro-Measurements, Raleigh, NC, USA), which is presented in Figure 2a. A constant value of Young’s modulus was used during the analysis, which equals 78 GPa. The measurements were made in 10 points located at different distances of the joint axis: two in the joint axis, two at the weld line, and six in the vicinity of the joint, both at the advancing side and retreating side. The schema of measuring point locations is presented in Figure 2b. Three measuring points had to be shifted from others during mounting in order to make the possibility of proper installation of rosettes.

Strains revealed during drilling and measured at the individual gauges of the rosette enabled determining of the values of principle stresses σ_max_ and σ_min_ together with their directions. Obtained results were used to calculate the values of stresses, longitudinal σ_11_, and transverse σ_22_, connected with the FSW joint. The error of computed values of residual stresses was estimated at ±(8–10) MPa in the whole range, which is acceptable. All tests mentioned in the experimental part were carried out 4 weeks after the joining process in order to stabilize the properties of the weld [2].

## 3. Results and Discussion

### 3.1. Macroscopic Observations

**Figure 3:**
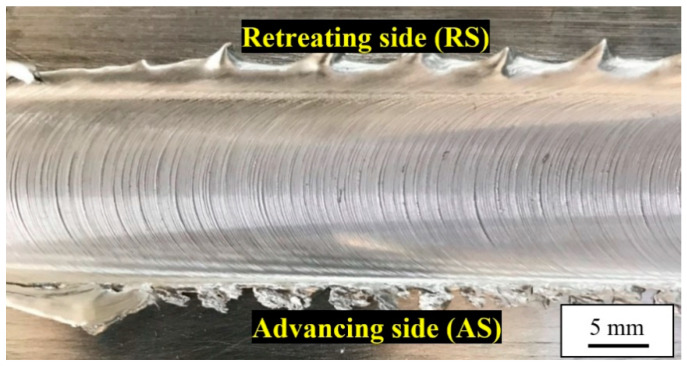
The weld face.

The weld face of the obtained joint is presented in Figure 3. For FSW joints, two sides of the weld can be distinguished: the retreating side (the direction of tool rotation is opposite to the welding direction) and the advancing side (the direction of tool rotation is concordant with the welding direction) [2]. The weld face is characterized by a typical, regular weld track, formed by the tool shoulder. As can be observed, no visible defects are present and the flash is localized mainly on the retreating side of the weld.

**Figure 4:**
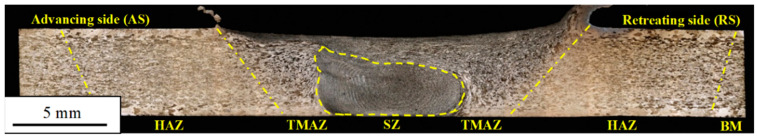
Macrostructure of the joint cross-section with distinguished zones.

The light microscopy image of the macroscopic view of the joint cross-section is presented in Figure 4. Lack of visible defects confirms that parameters used are appropriate for friction stir welding of this alloy. The width of the stir zone is about 8.5 mm. The welding process has reduced the thickness of the workpiece from 5 mm to 4.5 mm in the center of the weld. Generally, in FSW joint the two zones and one subzone can be distinguished: thermo-mechanically affected zone (TMAZ) formed due to effects of temperature and severe plastic deformation, stir zone (SZ), a specific subregion of TMAZ localized in the central part of a joint, characterized by the presence of ultrafine, dynamically recrystallized grains, and heat-affected zone (HAZ) affected only by the heat of the process. The observations revealed the presence of onion ring patterns in the stir zone, visible mainly at the advancing side of the weld. The differences in the macrostructure of the TMAZ/SZ boundaries at the advancing and retreating sides have been taken under investigation during microstructure analysis.

### 3.2. Microstructure Analysis and Microhardness Measurements

**Figure 5:**
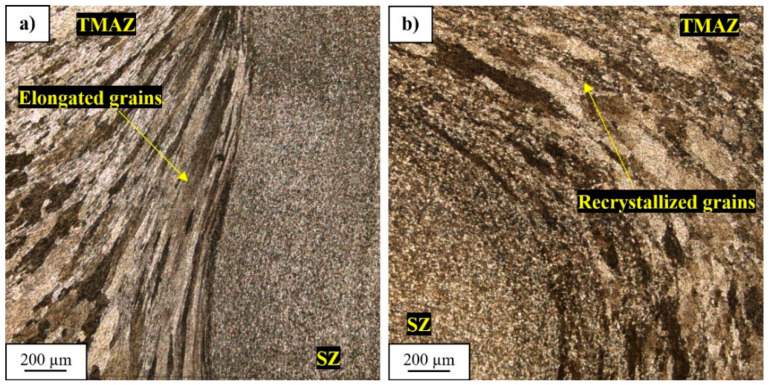
Microstructure of thermo-mechanically affected zone/stir zone (TMAZ/SZ) interface at the advancing side (**a**) and at the retreating side (**b**).

The microstructure of the TMAZ/SZ interface at the advancing side of the joint is characterized by the clear border between dynamically recrystallized grains of the stir zone and elongated grains of the thermo-mechanically affected zone, which visualize the flow of the material during the stirring process (Figure 5a). Concurrently, at the retreating side, the border between SZ and TMAZ is of transition nature (Figure 5b). The microstructure of TMAZ in close proximity to SZ can be identified as bands of recovered grains irregularly separated by recrystallized grains. These differences between each side have their source in the unsymmetrical distribution of the temperature during the FSW process. Studies revealed that the retreating side is characterized by higher effect of the temperature [40,41]. During welding, the generated heat influences the plastically deformed microstructure by promoting heat-activated phenomena such as grain recovery and recrystallization. As can be seen, the dynamic recovery has a predominant role while fine, dynamically recrystallized grains are localized mainly between large, deformed grains in the form of long bands (Figure 5b). This suggests that the grains which undergo dynamic recrystallization are compressed and intensely elongated by the larger grains in the stirring process and the quantity of heat allows them to rebuild their severely deformed microstructures. This phenomenon has not been observed at the advancing side (Figure 5a).

**Figure 6:**
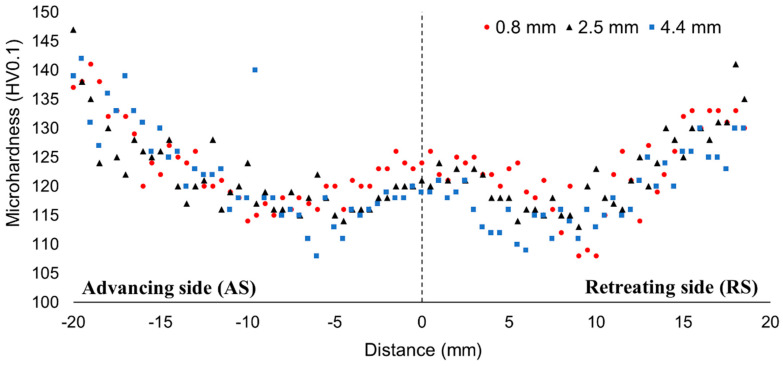
Microhardness profile of the welded joint cross-section.

The microhardness distribution of the join is presented in Figure 6. The obtained microhardness distribution allows observation of a typical “W”-shaped hardness curve. In the case of the advancing side, the lowest value of microhardness (108 HV0.1) was reported at the distance of 6 mm on the bottom part of the joint. Simultaneously, on the retreating side, the significant softening of welded material is reported. The low hardness zone is localized at the distance of 8 to 10 mm from the joint line, where the material was heated without being strain-hardened by plastic deformation. Generally, it can be stated that the reduction in microhardness predominantly affects the retreating side of the joint (about 25%), which confirms the higher effect of heat during welding in this area. Due to dynamic recrystallization in the stir zone leading to the formation of fine-grain microstructure, the microhardness slightly increases compared to TMAZ and HAZ. As can be seen, the highest value of microhardness in the stir zone is obtained for the upper part of this zone, where the effect of the tool shoulder severely refined the grains.

### 3.3. Tensile Test Results

**Figure 7:**
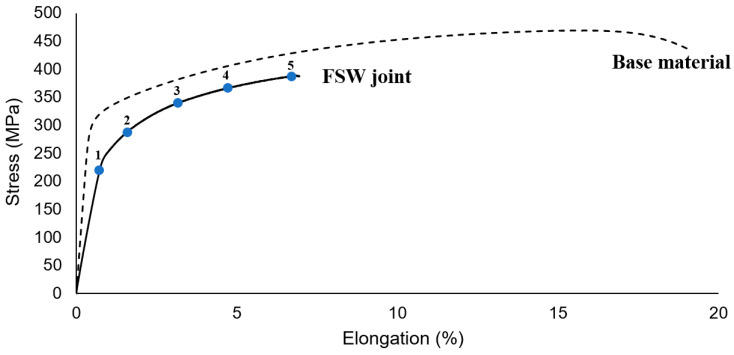
Engineering tensile stress–strain curves of base material and FSW joint.

The obtained tensile curves for AA2519-T62 base material and its friction stir welded joint are presented in Figure 7. The welding process has caused a significant reduction in elongation to failure of the material—from the value of 19% to 7%. The tensile strength of the tested joint was established as 389 MPa, which represents 83% joint efficiency. Comparing this value with the results of the studies [16,18], it is very high for high-strength aluminum alloy of 2XXX series, which can be partly explained by the high thickness of the workpiece (5 mm), which often results in the highest values of joint efficiency [2].

**Figure 8:**
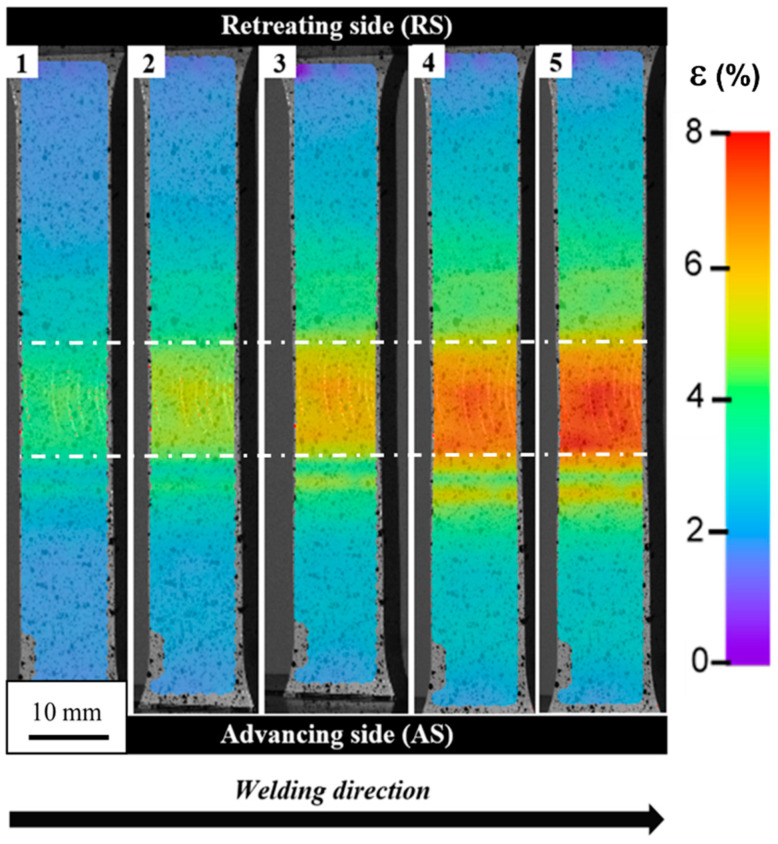
Strain distribution maps of the tensile sample corresponding to the points marked in Figure 7. Weld faces marked with white lines.

The digital image correlation technique provides more details about welded specimen behavior during the tensile test [42,43]. The obtained strain maps resulting from the DIC measurement for five points marked on the curve (Figure 7) are presented in Figure 8.

**Figure 9:**
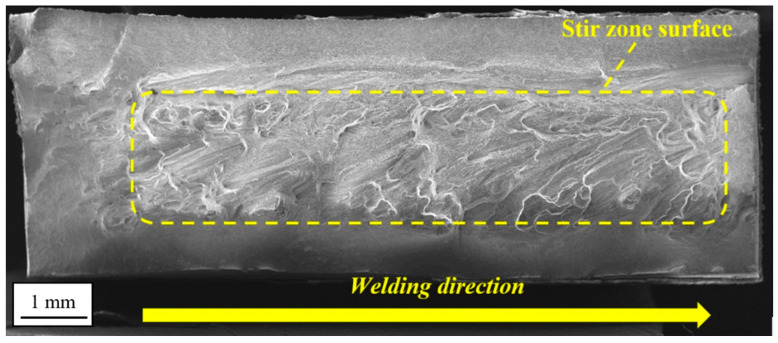
Fracture surface of FSW tensile sample.

The visualization of strain distribution allows observing of its uneven nature. The SZ and TMAZ are characterized by the highest strain, localized mainly in the central part of the SZ and advancing side of TMAZ. Of note is the fact that despite the retreating site the TMAZ has received a higher amount of heat during the welding process; its behavior in the tensile test reveals the spread of the strain without significant concentration in the specific area. The failure in the tensile test occurred in the TMAZ/SZ interface on the advancing side of the weld. The fracture surface of the specimen was subjected to scanning electron microscope observations (Figure 9).

The observations have revealed that decohesion took place on the boundary between SZ and TMAZ. This area was identified in the microstructure analysis part (Figure 5a) as consisting of severely deformed, elongated grains of TMAZ adjoining the dynamically recrystallized grains of SZ. Such differences in the microstructure promote decohesion and it can explain the better coherency of the SZ/TMAZ interface on the retreating side, where the boundary between each zone is not clear and TMAZ has partly recrystallized microstructure.

### 3.4. Residual Stress Measurements

In order to compute the residual stresses in the FSW joint the following equations were used [44], Equations (1)–(3):

(1) $$\sigma_{\max}=\;\frac{\varepsilon_{1}+{\;\varepsilon }_{3}}{4\;\cdot \;A}-\frac{1}{4\;\cdot \;B}\sqrt{{\left(\varepsilon_{3}-{\;\varepsilon }_{1}\right)}^{2}+\;\left(\varepsilon_{3}+\varepsilon_{1}-2\varepsilon_{2}\right){\;}^{2}}\;(\mathrm{MPa})$$

(2) $$\sigma_{\min}=\;\frac{\varepsilon_{1}+{\;\varepsilon }_{3}}{4\;\cdot \;A}+\frac{1}{4\;\cdot \;B}\sqrt{{\left(\varepsilon_{3}-{\;\varepsilon }_{1}\right)}^{2}+\;\left(\varepsilon_{3}+\varepsilon_{1}-2\varepsilon_{2}\right){\;}^{2}}\;(\mathrm{MPa})$$

(3) $$\alpha =\frac{1}{2}\mathrm{arctg}\frac{\varepsilon_{1}\;-\;2\varepsilon_{2}\;+{\;\varepsilon }_{3}}{\varepsilon_{3}-{\;\varepsilon }_{1}}$$

where: σ_max_ and σ_min_—the principle stresses; ε_1_, ε_2_, and ε_3_—the strains measured at the gauges of rosette no. 1, 2 and 3, respectively (Figure 2b); A and B—the coefficients dependent on material properties, type of rosette, and dimensions of drilled hole; and α—the angle between gauge no. 1 and the direction of the nearest principle stress.

**Figure 10:**
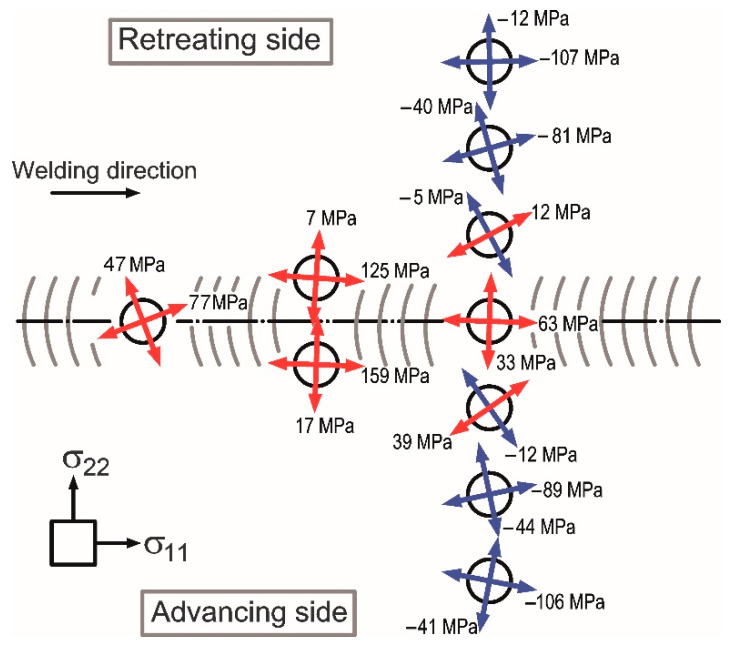
The directions and values of determined residual stresses in the FSW joint and its vicinity; σ_11_ and σ_22_ are the longitudinal and transverse stresses, respectively.

The obtained results are presented in graphical form in Figure 10. Red arrows indicate the directions of tension stresses and blue arrows indicate the directions of compression stresses.

**Figure 11:**
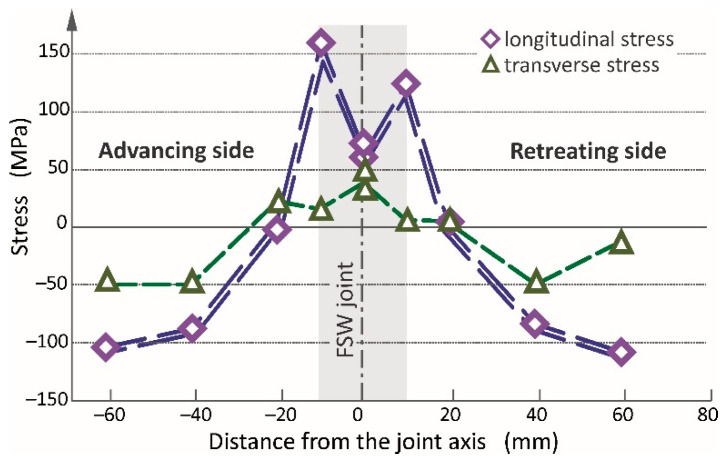
The distribution of longitudinal and transverse stresses in the FSW joint and its vicinity.

Results obtained by hole-drilling method were used to compute the values of longitudinal stresses σ_11_ and transverse stresses σ_22_. Nevertheless, in most cases, the values were obtained directly during the measurements because the directions of the main gauges (tensometers nos. 1 and 3) were convergent with the directions of searched stresses. The Equation (4) was used in other cases.

(4) $$\sigma_{11\left(22\right)}=\frac{\sigma_{\max\left(\min\right)}\;+{\;\sigma }_{\min\left(\max\right)}}{2}+\frac{\sigma_{\max\left(\min\right)}\;-{\;\sigma }_{\min\left(\max\right)}}{2}\cdot \cos2\alpha$$

where α is an angle between the directions of searched stress σ_11(22)_ and known principle stress σ_max(min)_. The results are presented in Figure 11.

The results shown in Figure 11 indicate that tension stresses are present in the joint and its nearest vicinity. Greater values of stresses are in a longitudinal direction than transverse; additionally, they are greater on the advancing side than the retreating side. As the retreating side is characterized by higher heat input, it promotes stress relaxation and can explain the lower value of residual stress in this zone of the weld [40,41]. The highest stresses are at the weld line on the advancing side (159 MPa). The stresses in a joint center reach of 33–77 MPa and are similar in all directions. At the distance of approximately 10 mm from the weld joint, the stresses have very low values, near to zero. It should be noted that at these points the direction of principle stresses is the most rotated of all. It is probably caused by the material flow during the stirring process resulting in the plastic deformation of less heated areas in the surrounding area. With increasing distance from the joint, the stresses change to compressive which are significantly greater in a longitudinal direction contrary to transverse. The obtained values of residual stresses and their nature of changes are similar to those available in other publications [45,46,47,48,49,50]. In all cases, the residual stresses are the highest at the weld line and lower at the center of joints. The values of stresses significantly decrease and change from tension stresses into compression. Moreover, the maximum value of measured residual stress equals approximately 50% of the yield stress of paternal material AA2519-T62. It should be noted that during the welding using either TIG or laser method the residual stresses can even reach the yield stress [2,3,51].

### 3.5. Low Cycle Fatigue Properties and Fracture Surface Observations

**Figure 12:**
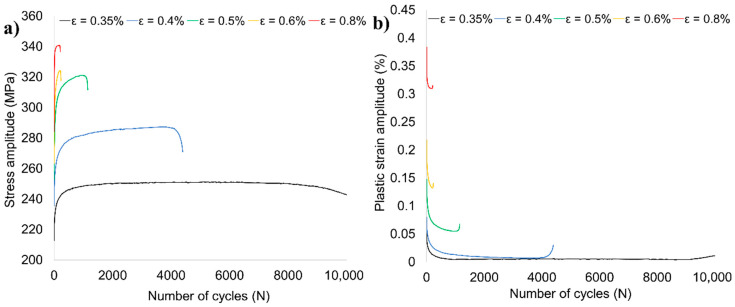
Variation of stress amplitude (**a**) and plastic strain amplitude (**b**) with the number of cycles.

The variations of stress and plastic strain amplitudes with the number of cycles are shown in Figure 12a,b respectively. Regardless of the strain amplitude, three periods of fatigue life can be distinguished: cyclic hardening, cyclic stabilization, and final rapid drop in the value of stress amplitude until failure, which corresponds to the cyclic properties of the base material [52]. The length of each period differs depending on the used strain amplitude. Especially, for the highest values of strain amplitude (0.5, 0.6, and, 0.8%), it is difficult to identify the cyclic stabilization period and the samples undergo cyclic hardening until rapid failure. Compared to the base material, the obtained values of stabilized stress amplitude (Figure 12a) are lower for about 25% and also the reduction of fatigue life was reported [52]. At the same time, the registered plastic strain amplitudes of the AA2519-T62 FSW joint are much higher (Figure 12b). These differences can be partly explained by the lower ductility of the obtained joint compared to the base material (Figure 7).

**Figure 13:**
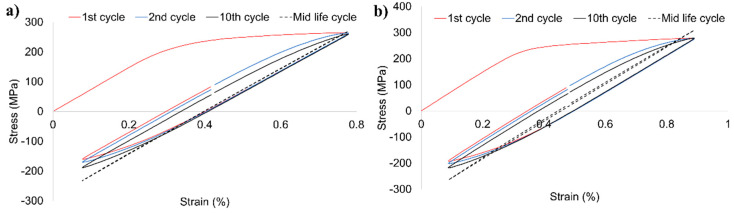

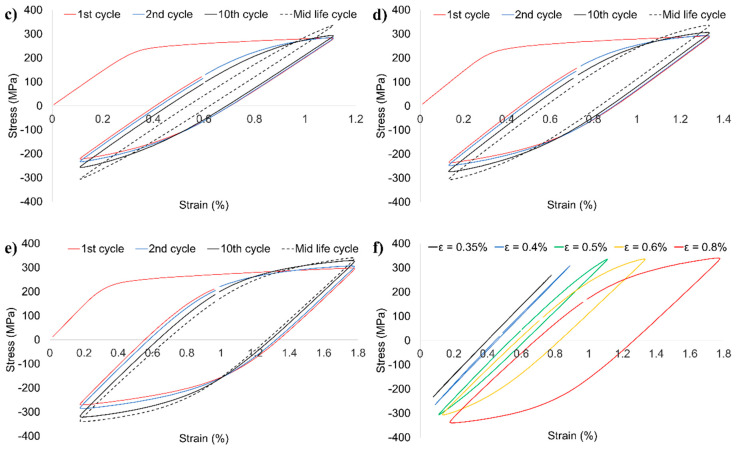
Stress–strain loops of FSW joint tested with ε = 0.35% (**a**), ε = 0.4% (**b**), ε = 0.5% (**c**), ε = 0.6% (**d**), ε = 0.8% (**e**), and the comparison of mid-life loops (**f**).

The hysteresis stress–strain loops of examined welded joint for various levels of strain amplitude are presented in Figure 13a–e and the stabilized loops are compared in Figure 13f.

**Figure 14:**
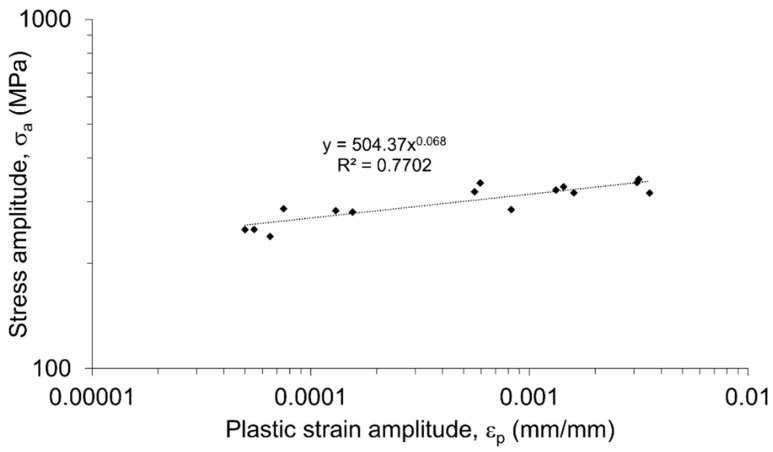
Stress amplitude versus plastic strain amplitude of FSW joint stabilized hysteresis loops in log-log coordinates.

The obtained loops reflect the fatigue properties observed in Figure 12a,b. In the first cycles of fatigue life, cyclic hardening occurs, which is much more visible in the samples tested with the strain amplitudes of 0.5, 0.6, and 0.8% (Figure 13c–e). The same strain amplitudes are characterized by a noticeable dissipation of strain energy manifested in a relatively high width of the mid-life hysteresis loops. The dissipation, caused predominantly by the plastic deformation leading to the development of microcracks [53], increases significantly together with the applied strain amplitude (Figure 13f). The parameters of the stabilized loops allowed establishing of the relationship of stress amplitude versus plastic strain amplitude, presented in Figure 14.

The obtained curve can be described by the following equation [54]:

(5) $$\sigma_{a}=\;k\prime {\left(\varepsilon_{p}\right)}^{\;n\prime }$$

where: σ_a_—stress amplitude (MPa), ε_p_—plastic strain amplitude [mm/mm], k′—cyclic strength coefficient (MPa), and n′—cyclic strain hardening exponent. The values of k’ and n’ have been taken directly from the function describing the plot in Figure 14:

(6) $$\sigma_{a}=504.37{\left(\varepsilon_{p}\right)}^{0.068}$$

**Figure 15:**
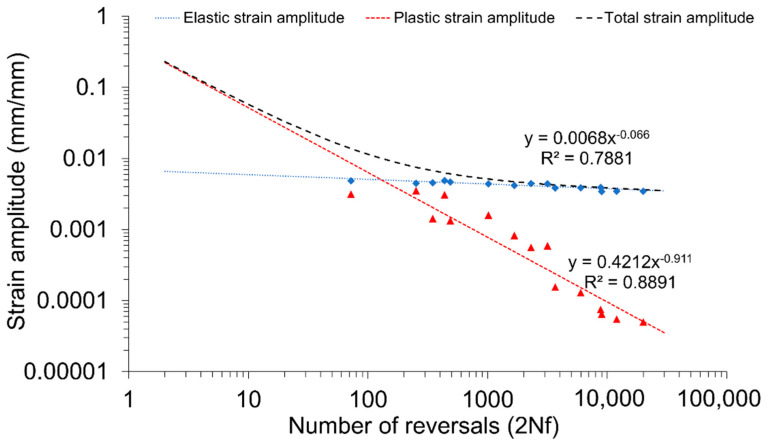
Number of half-cycle reversals vs. strain amplitude in log-log coordinates.

The obtained value of the cyclic strength coefficient (504.37 MPa) is almost three times lower than for the base material (1518.1 MPa) [52]. Although, the decrease of cyclic strength coefficient is expected for welded joints, in this case, the reported drop is significant and the obtained value is similar to the values reported by research [31,36,55,56,57]. In the next step, the parameters of the stabilized loops were used for establishing the plots of elastic and plastic strain amplitudes vs. number of reversals, presented in Figure 15.

The Manson–Coffin–Basquin equation is described by the following formula [54]:

(7) $$\varepsilon =\varepsilon_{e}+{\;\varepsilon }_{p}=\;\frac{\sigma_{f}^{\prime }}{E}{\left(2N_{f}\right)}^{b}+{\;\varepsilon \prime }_{f}{\left(2N_{f}\right)}^{c}$$

where: σ′_f_—fatigue strength coefficient (MPa), E—Young modulus (MPa); b—fatigue strength exponent; ε′_f_—fatigue ductility coefficient; and c—fatigue ductility exponent. The value of Young modulus of the welded joint was established in the tensile test and it is equal to 68 GPa. The specific values have been taken from the functions describing the plots in Figure 15:

(8) $$\varepsilon =\varepsilon_{e}+{\;\varepsilon }_{p}=\;\frac{462.4}{68,000}{\left(2N_{f}\right)}^{-0.066}+\;0.4212\;{\left(2N_{f}\right)}^{-0.911}$$

In terms of the plastic strain component, it is very close to the parameters set up for base material [52], while the elastic strain component deviates from it noticeably.

**Figure 16:**
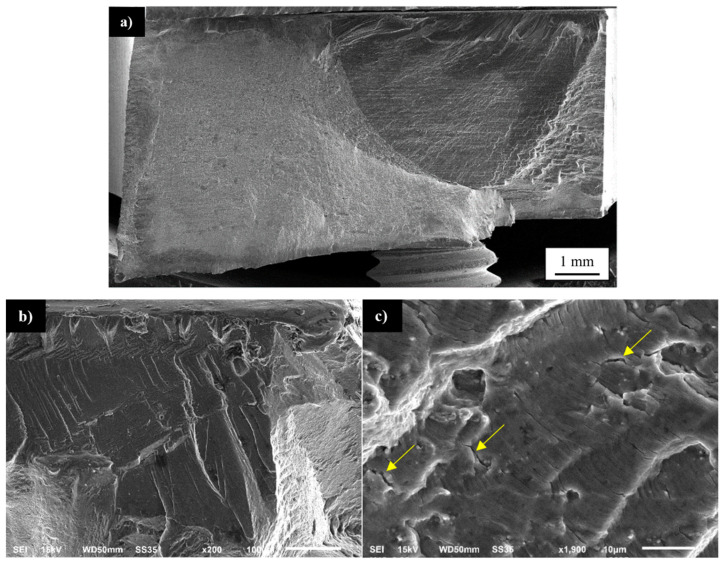
SEM images of fatigue surfaces of a specimen tested at a strain amplitude of 0.35%: (**a**) overall view of the fracture surface, (**b**) magnified view of the crack initiation zone, and (**c**) propagation region near the initiation site. The yellow arrows indicate the secondary cracks.

The tested samples tend to fail in the HAZ on the retreating side of the joint and for the lowest value of strain amplitude (0.35%) and in the case of the higher values of amplitude the failure occurred at the boundary of TMAZ/SZ on the advancing side of the weld. This zone was characterized in the microstructure analysis part (Figure 5a) and it is also a place of failure in the tensile test (Figure 9). For the fracture surface observations, the three representative samples were selected and tested at the amplitudes of 0.35%, 0.5%, and 0.8%. The fracture surface of the first sample is presented in Figure 16a–c.

**Figure 17:**
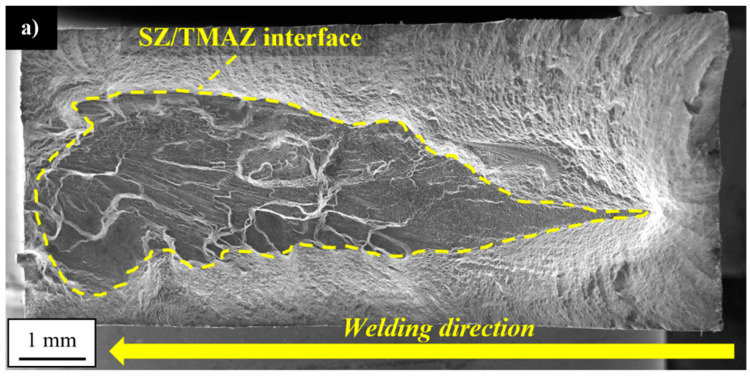

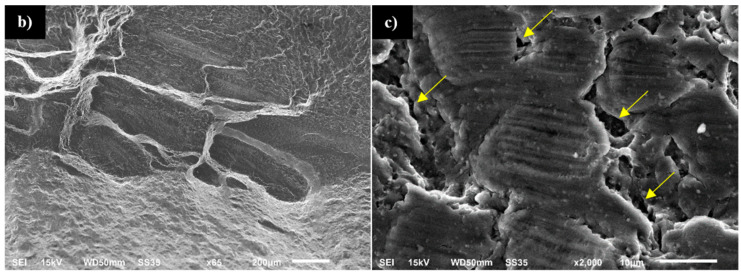
SEM images of fatigue surfaces of a specimen tested at a strain amplitude of 0.5%: (**a**) overall view of the fracture surface, (**b**) bottom part of the fracture surface, and (**c**) fatigue striations. The yellow arrows indicate the secondary cracks.

The fracture surface has a typical structure of fatigue fracture, consisting of an initiation site, fatigue crack propagation region, and rapid fracture area (Figure 16a). The initiation of fatigue crack in localized in the top corner of the investigated sample, the surface is free of visible defects, smooth, and contains river-like patterns (Figure 16b). The fatigue crack propagation area is characterized by the presence of fatigue striations with noticeable participation of secondary cracks (marked with yellow arrows) indicating a local intensification of material decohesion (Figure 16c). The observed fractured surface is of mixed ductile and brittle character. The fracture surface of the sample tested with the strain amplitude of 0.5% is presented in Figure 17a–c.

The surface is more similar to the tensile sample and no areas typical for fatigue fracture have been distinguished under macroscopic observation. The observed surface contains part of the SZ/TMAZ interface with a visible texture demonstrating plastic flow in the stirring process (Figure 17a). At this interface, it is possible to identify vertical “cliffs” suggesting the grain-boundary separation (Figure 17b). However, the higher magnification revealed the fatigue striations and occurrence of numerous, very deep secondary cracks (Figure 17c). Not only the specific microstructure (Figure 5a) but also the high values of residual stresses (Figure 11) can promote decohesion in this area. The near-surface stresses directly influence the initiation of a fatigue crack. This phenomenon is of great importance in the cases of relatively low plastic strain amplitudes, and for the large plastic deformation, the residual stresses tend to relax.

**Figure 18:**
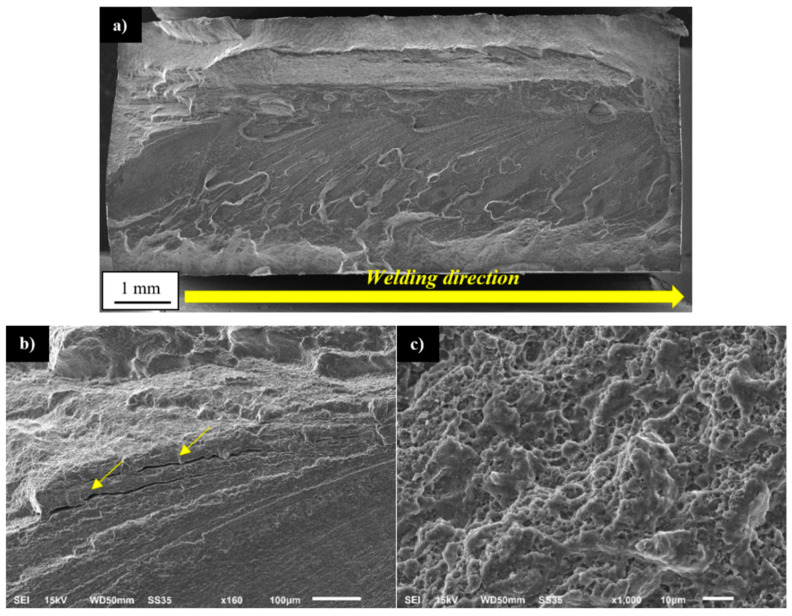
SEM images of fatigue surfaces of a specimen tested at a strain amplitude of 0.8%: (**a**) overall view of the fracture surface, (**b**) upper part of the SZ surface, and (**c**) magnified view of the SZ surface. The yellow arrows indicate the secondary cracks.

No noticeable presence of secondary cracks between the fatigue striations was reported. The fracture surface of the sample tested with the strain amplitude of 0.8% is presented in Figure 18a–c.

The character of failure is similar to the quasi-static fracture (Figure 9) with high participation of the SZ surface (Figure 18a) and no reported presence of fatigue striations (Figure 18a). In the upper part of the SZ interface the occurrence of secondary cracks was registered (Figure 18b), probably the delamination of compressed, elongated grains of the TMAZ, which have been identified in this zone during the microstructure analysis (Figure 5a). The higher magnification of the SZ surface revealed the typical, fine dimple structure (Figure 18c) with very small precipitates (below 1 μm).

## 4. Conclusions

The performed research on the mechanical properties and low cycle fatigue of friction stir welded Sc-modified AA2519-T62 allowed drawing the following conclusions:

1. The microstructure analysis of the obtained weld has revealed the differences in the boundaries between SZ and TMAZ on each side of the joint. On the advancing side, the severely deformed and elongated grains of TMAZ are adjoined to the dynamically recrystallized grains of SZ, while on the retreating side the boundary between SZ and TMAZ is not clear and TMAZ is characterized by partly recrystallized microstructure.
2. The failure in the tensile test has occurred in the boundary of TMAZ/SZ on the advancing side of the weld with the registered value of 83% joint efficiency. The changes in strain distribution in the FSW joint zone on the local scale were successfully assessed by the DIC system.
3. The residual stress measurements have revealed that the highest values of longitudinal stress are localized at the distance of 10 mm from the joint line with their values of 124 MPa (the retreating side) and 159 MPa (the advancing side).
4. The cyclic hardening of AA2519-T62 FSW joints during the low-cycle fatigue process were revealed by a reduction of the width of hysteresis loops during the test, with the simultaneous increase in the range of stress values.

## Figures and Tables

**Table 1:** Chemical composition of AA2519 (% weight) [34].

Fe	Si	Cu	Zn	Ti	Mn	Mg	Ni	Zr	Sc	V	Al
0.11	0.08	6.32	0.05	0.08	0.17	0.33	0.02	0.19	0.16	0.10	Base

**Table 2:** Mechanical properties of AA2519-T62 [34].

Young Modulus (E)	Yield Strength (R_e0.2_)	Tensile Strength (R_m_)	Elongation (A)
78 GPa	312 MPa	469 MPa	19%
